# A machine learning model based on results of a comprehensive radiological evaluation can predict the prognosis of basal ganglia cerebral hemorrhage treated with neuroendoscopy

**DOI:** 10.3389/fneur.2024.1406271

**Published:** 2024-10-01

**Authors:** Xiaolong Hu, Peng Deng, Mian Ma, Xiaoyu Tang, Jinghong Qian, YuHui Gong, Jiandong Wu, Xiaowen Xu, Zhiliang Ding

**Affiliations:** ^1^Department of Neurosurgery, The Affiliated Suzhou Hospital of Nanjing Medical University, Suzhou Municipal Hospital, Gusu School of Nanjing Medical University, Suzhou, China; ^2^Department of Emergency and Critical Care Medicine, The Affiliated Suzhou Hospital of Nanjing Medical University, Suzhou Municipal Hospital, Gusu School of Nanjing Medical University, Suzhou, China

**Keywords:** basal ganglia hemorrhage, machine learning model, radiomics, prognosis, neuroendoscopy

## Abstract

**Introduction:**

Spontaneous intracerebral hemorrhage is the second most common subtype of stroke. Therefore, this study aimed to investigate the risk factors affecting the prognosis of patients with basal ganglia cerebral hemorrhage after neuroendoscopy.

**Methods:**

Between January 2020 and January 2024, 130 patients with basal ganglia cerebral hemorrhage who underwent neuroendoscopy were recruited from two independent centers. We split this dataset into training (*n* = 79), internal validation (*n* = 22), and external validation (*n* = 29) sets. The least absolute shrinkage and selection operator-regression algorithm was used to select the top 10 important radiomic features of different regions (perioperative hemorrhage area [PRH], perioperative surround area [PRS], postoperative hemorrhage area [PSH], and postoperative edema area [PSE]). The black hole, island, blend, and swirl signs were evaluated. The top 10 radiomic features and 4 radiological features were combined to construct the k-nearest neighbor classification (KNN), logistic regression (LR), and support vector machine (SVM) models. Finally, the performance of the perioperative hemorrhage and postoperative edema machine learning models was validated using another independent dataset (*n* = 29). The primary outcome is mRS at 6 months after discharge. The mRS score greater than 3 defined as functional independence.

**Results:**

A total of 12 models were built: PRH-KNN, PRH-LR, PRH-SVM, PRS-KNN, PRS-LR, PRS-SVM, PSH-KNN, PSH-LR, PSH-SVM, PSE-KNN, PSE-LR, and PSE-SVM, with corresponding areas under the curve (AUC) values in the internal validation set of 0.95, 0.91, 0.94, 0.52, 0.91, 0.54, 0.67, 0.9, 0.72, 0.92, 0.92, and 0.95, respectively. The AUC values of the PRH-KNN, PRH-LR, PRH-SVM, PSE-KNN, PSE-LR, and PSE-SVM in the external validation were 0.9, 0.92, 0.89, 0.91, 0.92, and 0.88, respectively.

**Conclusion:**

The model built based on computed tomography images of different regions accurately predicted the prognosis of patients with basal ganglia cerebral hemorrhage treated with neuroendoscopy. The models built based on the preoperative hematoma area and postoperative edema area showed excellent predictive efficacy in external verification, which has important clinical significance.

## 1 Introduction

Spontaneous intracerebral hemorrhage (ICH) is the second most common subtype of stroke, with a fatality rate of 35–52% within 30 days of onset. The basal ganglia are the most common sites of ICH; more than 70% of patients with basal ganglia cerebral hemorrhage have functional dependence or even die (1, 2). Currently, the main surgical methods for treating basal ganglia cerebral hemorrhage are traditional craniotomy hematoma removal and minimally invasive neuroendoscopic hematoma removal. Multiple studies have shown that minimally invasive endoscopic hematoma evacuation offers greater prognostic benefits for patients than traditional craniotomy hematoma evacuation (3), and it is widely applied in clinical practice. However, analyzing the prognostic risk factors for endoscopic treatment of basal ganglia cerebral hemorrhage and constructing a prognostic prediction model to assist in clinical decision-making are of paramount importance.

The island (4), swirl (5), and blend signs (6, 7) are special radiological manifestations observed on computed tomography (CT) scans of patients with ICH. Their appearance can accurately predict hematoma expansion and a poor prognosis. Recent studies (8, 9) have shown that the volume of ICH and postoperative edema are closely related to the postoperative functional dependency of patients (modified Rankin Scale [mRS] score, >3). Heterogeneity exists in hematoma and edema, which contain a wealth of information that is closely related to patient prognosis.

Radiomics (10, 11) is a new image analysis technology that has emerged in recent years. It can transform images into high-throughput quantitative data, including first-order features and gray-level features, to reveal target information. Radiomics is expected to help explore new prognostic imaging markers to assist in clinical decision-making.

Machine learning is a crucial component within the realm of artificial intelligence. Its application is instrumental in facilitating the handling of high-throughput data, thereby enabling thorough analysis and discernment of patterns indicative of disease features.

Hence, this study employed radiomics techniques to extract CT imaging features from different regions of patients with basal ganglia cerebral hemorrhage to investigate the correlation between radiomics features and long-term functional dependency after discharge. In addition, a prognostic prediction model was developed to provide guidance for clinical practice.

## 2 Materials and methods

### 2.1 Ethics statement

This study was approved by the hospital’s Ethics Review Committee, and informed consent was obtained from all patients.

### 2.2 Study design and population

**Figure 1:**
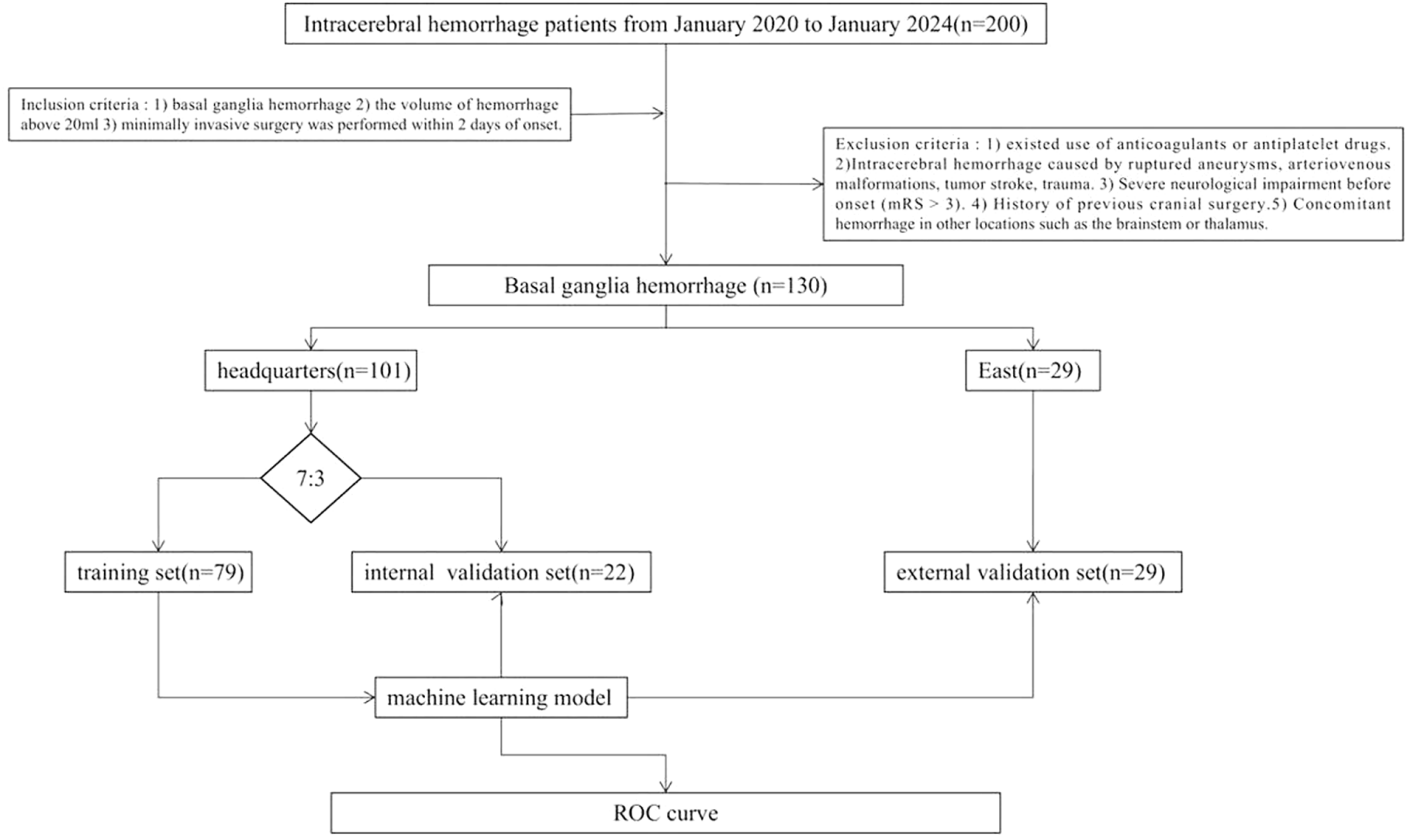
Flowchart of study design showing selection of cases along with exclusion criteria.

This study retrospectively included 230 patients with medium-vessel occlusion treated endoscopically at Suzhou Municipal Hospital East District (*n* = 29) and Suzhou Municipal Hospital Headquarters (*n* = 101) between January 2020 and January 2024. The patients were divided into training (*n* = 22), internal validation (*n* = 79), and external validation (*n* = 29) sets. The inclusion criteria were as follows: (1) basal ganglia cerebral hemorrhage, (2) volume of hemorrhage >20 mL, and (3) minimally invasive surgery performed within 2 days of onset. The exclusion criteria were as follows: (1) use of anticoagulants or antiplatelet drugs; (2) ICH caused by ruptured aneurysms, arteriovenous malformations, tumor stroke, or trauma; (3) severe neurological impairment before onset (mRS score, >3); (4) history of previous cranial surgery; and (5) concomitant hemorrhage in other locations, e.g., the brainstem or thalamus. Figure 1 shows the study flow chart.

### 2.3 Minimally invasive endoscopic treatment of basal ganglia cerebral hemorrhage

All enrolled patients completed routine preoperative examinations, such as head CT (Philips Medical Systems, Cleveland), and head CT image data were reconstructed using 3D Slicer (version 4.9.0; National Institutes of Health) to delineate the hematoma. Pre-designed markers were placed on the patient’s scalp on the basis of the location and anatomical position of the hematoma. Depending on the requirement to minimize the distance between the hematoma and cortex and avoid functional areas, either the transcortical approach through the frontal lobe or the insular/lateral sulcus approach was chosen. A 3-cm incision was made on the scalp by the surgeon, followed by exposure of the skin, subcutaneous tissue, and skull. A skull perforation was created using a pneumatic drill to create a small hole with a diameter of 2–3 cm. The endocranium was coagulated and cross-cut using bipolar electrocoagulation, and a puncture needle was used to puncture the hematoma cavity. After removing the needle core, the syringe was withdrawn to reduce the intracranial pressure. An endoport transparent sheath was placed, and a 0° endoscope was introduced into the sheath to provide a surgical field of view. Then, the hematoma was gradually aspirated from deep to shallow depths using a microsuction device. Hematoma removal was achieved using bipolar electrocoagulation and a gelatin sponge under endoscopic guidance. After clearing the hematoma under microscopy, the sheath was slowly withdrawn, the hematoma removal was completed, and the skull was closed layer-by-layer.

### 2.4 ROI (region of interest) segementation, radiomics features extraction and selection

**Figure 2:**
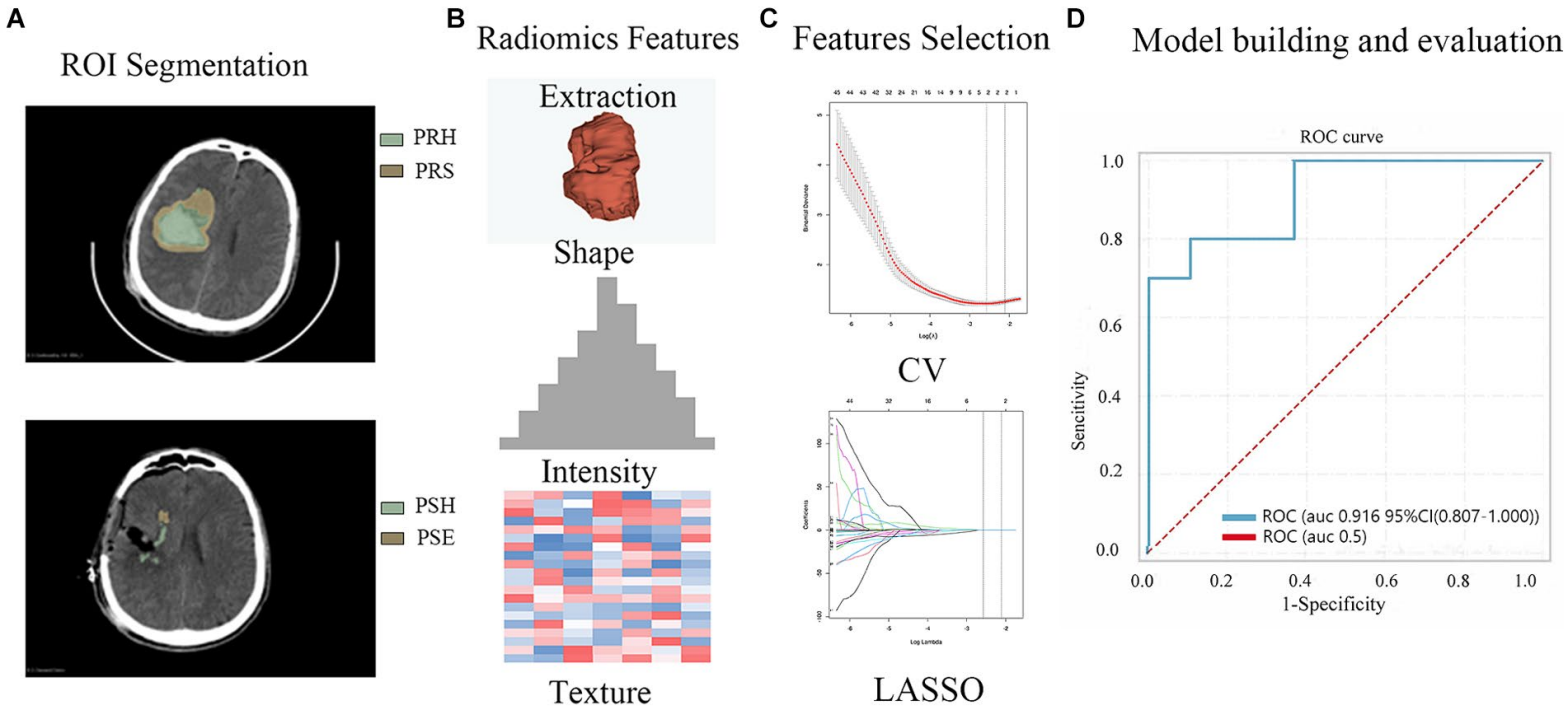
The workflow of the radiomics feature extraction, model building and evaluation process. **(A)** ROI (Region of Interest, PRH, PRS, PSH PSE) Segmentation **(B)** Radiomics Features Extraction **(C)** Feature Selection **(D)** Model building and evaluation (PRH-LR model in the validation set).

All patients underwent at least one preoperative and postoperative head CT scan (Philips) with a slice thickness of 5 mm. Using 3D Slicer software (version 4.9.0; National Institutes of Health), the preoperative hematoma area (PRH), perioperative surround area (PRS; a 5-mm region surrounding the hematoma with high-density shadows on the CT brain tissue window), postoperative edema area (PSE; appearing as low-density shadows on the CT brain tissue window), and postoperative hemorrhage area (PSH) were delineated layer-by-layer on CT transverse sections (Figure 2A). Subsequently, radiomics features were automatically extracted using pyradiomics (12) (Figure 2B) and maintained with voxel resampling at 1 × 1 × 1 mm. In total, 107 features were extracted from each region, including histogram features, morphological features, gray-level co-occurrence matrices (GLCMs), gray-level size zone matrices (GLSZMs), gray-level run length matrices (GLRLMs), neighboring gray-tone difference matrices (NGTDMs), and gray-level dependence matrices (GLDMs). The aforementioned procedures were independently repeated by two neurosurgeons with 10 years of experience. The intraclass correlation coefficient (ICC) was used to assess the consistency of radiomic features. The least absolute shrinkage and selection operator (LASSO) regression algorithms were used to rank the importance of the variables (Figure 2C). The 10 most important variables were used to build the model.

### 2.5 Radiological feature evaluation

On the basis of head CT findings, the black hole (13), island (4), blend (6, 7), and swirl signs (5) were individually assessed by two neurosurgeons, each with 10 years of work experience. If there was a discrepancy, a third neurosurgeon with higher seniority made the final decision.

### 2.6 Clinical outcome

The mRS was used to assess functional outcomes at 6 months after discharge. An mRS score of >3 indicates functional dependency; otherwise, an mRS score of <3 indicates functional independence.

### 2.7 Training and validation of the machine learning models

Radiological and radiomic features of each region were used to construct machine learning models (k-nearest neighbor classification [KNN], logistic regression [LR], and support vector machine [SVM]). Twelve models were constructed: PRH-KNN, PRH-LR, PRH-SVM, PRS-KNN, PRS-LR, PRS-SVM, PSH-KNN, PSH-LR, PSH-SVM, PSE-KNN, PSE-LR, and PSE-SVM. The datasets from the Suzhou Municipal Hospital Headquarters (*n* = 101) were randomly divided into training and internal validation sets at a ratio of 7:3. The datasets from the Suzhou Municipal Hospital East District were considered as the external validation set. The Receiver Operating Characteristic Curve (ROC) used to test the model.

### 2.8 Statistical analyses

Normally distributed continuous data are presented as mean ± standard deviation. The independent samples *t*-test was used to compare data between the groups. For non-normally distributed continuous data, the median, first quartile (Q1), and third quartile (Q3) are reported, and group comparisons were conducted using the Mann–Whitney U test. Count data are presented as frequency (percentage) [*n*, %], and group comparisons were assessed using the chi-square test. The Fisher exact test was used to determine between-group differences when the theoretical cell frequency was <5. Receiver operating characteristic curve analysis was used to evaluate the predictive performance of various models for postoperative functional dependency. The area under the curve (AUC), sensitivity, specificity, and accuracy values were calculated. Statistical analyses were performed using RStudio software (version 4.3.1; Posit). Statistical significance was defined as *p* < 0.05.

## 3 Results

### 3.1 Patients’ characteristics

Table 1 shows the baseline characteristics of patients with basal ganglia cerebral hemorrhage at the Suzhou Municipal Hospital Headquarters (*n* = 101). The baseline characteristics of the training and internal validation sets were not significantly different (*p* > 0.05).

**Table 1:** Patient’s baseline characteristics.

Clinical characteristics	Functional independency (*n* = 36)			Functional dependency (*n* = 65)			Test value	*p*-value
Sex						0.85	0.36	
Male		29			47			
Female		7			18			
Smoke							0.43	0.51
Yes		8			11			
No		28			54			
Diabetes							2.4	0.12
Yes		8			7			
No		28			58			
Hypertension							0.02	0.89
Yes		25			46			
No		11			19			
Age, y	55.89 ± 13.76			58.13 ± 13.19			0.82	0.61
GCS score	11.72 ± 2.72			8.69 ± 3.04			4.97	0.19

GCS, Glasgow Coma Scale.

### 3.2 Radiomics and radiological features selection

Out of the 428 radiomics features, 408 (95%) had ICCs larger than 0.9, indicating good consistency of the data. The top 10 radiomic features were selected for each region using the LASSO regression algorithm (Figure 1). Table 2 lists the radiomics features used to build the model. Table 3 shows the patients’ radiological features.

**Table 2:** Variations in the model.

Model	Radiomics features	Respective importance value
PRH-KNN, PRH-LR, PRH-SVM	gldmLowGrayLevelEmphasis, glcmMCC, shapeFlatness, shapeElongation, glcmImc1, firstorderEntropy, glcmJointEnergy, shapeSurfaceVolumeRatio, glcmDifferenceEntropy, glszmSizeZoneNonUniformityNormalized	20.44, 9.99, 6.00, 5.97, 4.77, 3.99, 3.52, 3.36, 2.68, 2.39
PRS-KNN, PRS-LR, PRS-SVM	glrlmLongRunLowGrayLevelEmphasis, firstorderInterquartileRange, ngtdmComplexity, firstorder10Percentile, shapeMaximum2DDiameterColumn, firstorderMedian, glszmSmallAreaHighGrayLevelEmphasis, shapeMaximum2DDiameterRow, glrlmLongRunHighGrayLevelEmphasis, shapeMaximum3DDiameter	0.22, 0.07, 0.03, 0.03, 0.03, 0.03, 0.02, 0.02, 0.01, 0.01
PSH-KNN, PSH-LR, PSH-SVM	glszmZoneEntropy, firstorder90Percentile, firstorderMedian, glrlmLongRunLowGrayLevelEmphasis, firstorderKurtosis, firstorderInterquartileRange, firstorder10Percentile, glrlmLongRunEmphasis, glszmHighGrayLevelZoneEmphasis, shapeMaximum2DDiameterSlice	0.16, 0.11, 0.06, 0.06, 0.06, 0.03, 0.03, 0.03, 0.02, 0.02, 0.02
PSE-KNN, PSE-LR, PSE-SVM	gldmSmallDependenceLowGrayLevelEmphasis, glcmDifferenceVariance, gldmGrayLevelVariance, glrlmGrayLevelNonUniformityNormalized, glcmCorrelation, glrlmRunLengthNonUniformityNormalized, ngtdmCoarseness, glcmImc2, glrlmShortRunLowGrayLevelEmphasis, glcmIdn	128.41, 11.85, 10.65, 10.60, 8.69, 8.27, 6.70, 5.61, 5.55, 5.07

PRH, perioperative hemorrhage area; PRS, perioperative surround area; PSH, postoperative hemorrhage area; PSE, postoperative edema area; KNN, k-nearest neighbor classification; LR, logistic regression; SVM, support vector machine.

**Table 3:** Patients’ radiological features.

Radiological features	Functional independency (*n* = 36)			Functional dependency (*n* = 65)			Test value	*p*-value
Island sign							35.47	<0.01
Yes		9			55			
No		27			10			
Black hole							28.97	<0.01
Yes		13			57			
No		23			8			
Blend sign							24.77	<0.01
Yes		13			55			
No		23			10			
Swirl sign							28.97	<0.01
Yes		13			57			
No		23			8			

### 3.3 Comprehensive radiological model constructed using the machine learning methods

**Figure 3:**
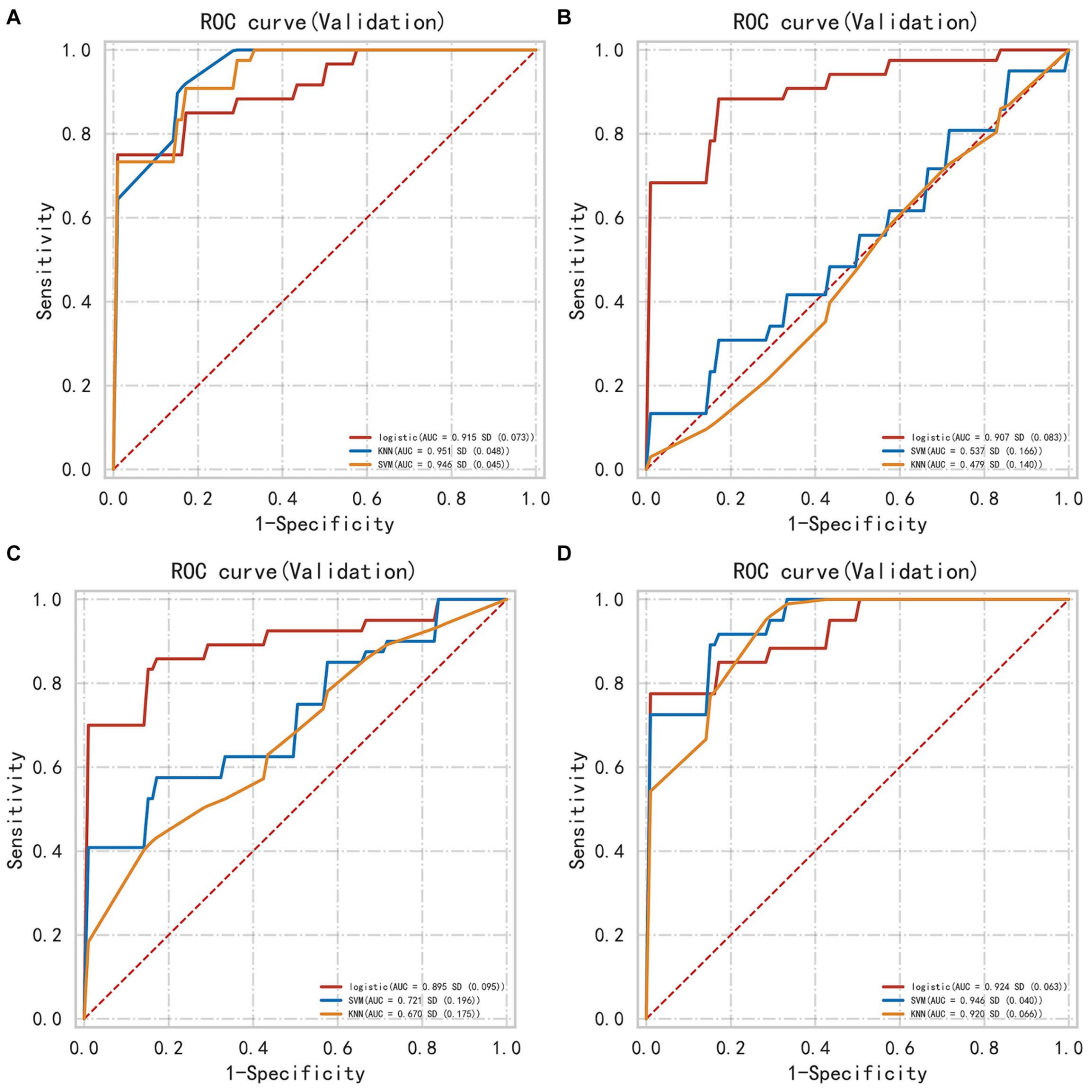
The AUC of different models evaluated in internal validation data **(A)** perioperative model **(B)** perioperative surround model **(C)** postoperative hemorrhage model **(D)** postoperative edema model. This figure illustrates the Area Under the Curve (AUC) for different machine learning models, including KNN, SVM, and LR, evaluated on internal validation datasets. The AUC is calculated for each model based on features extracted from distinct anatomical regions. Notably, KNN and SVM models exhibit substantial variability in their AUC values across different regions, indicating their sensitivity to regional feature sets. In contrast, the LR model demonstrates a more consistent performance, maintaining relatively stable AUC values regardless of the region from which features are derived. This suggests that LR may be more robust in handling varying feature distributions across different regions.

**Figure 4:**
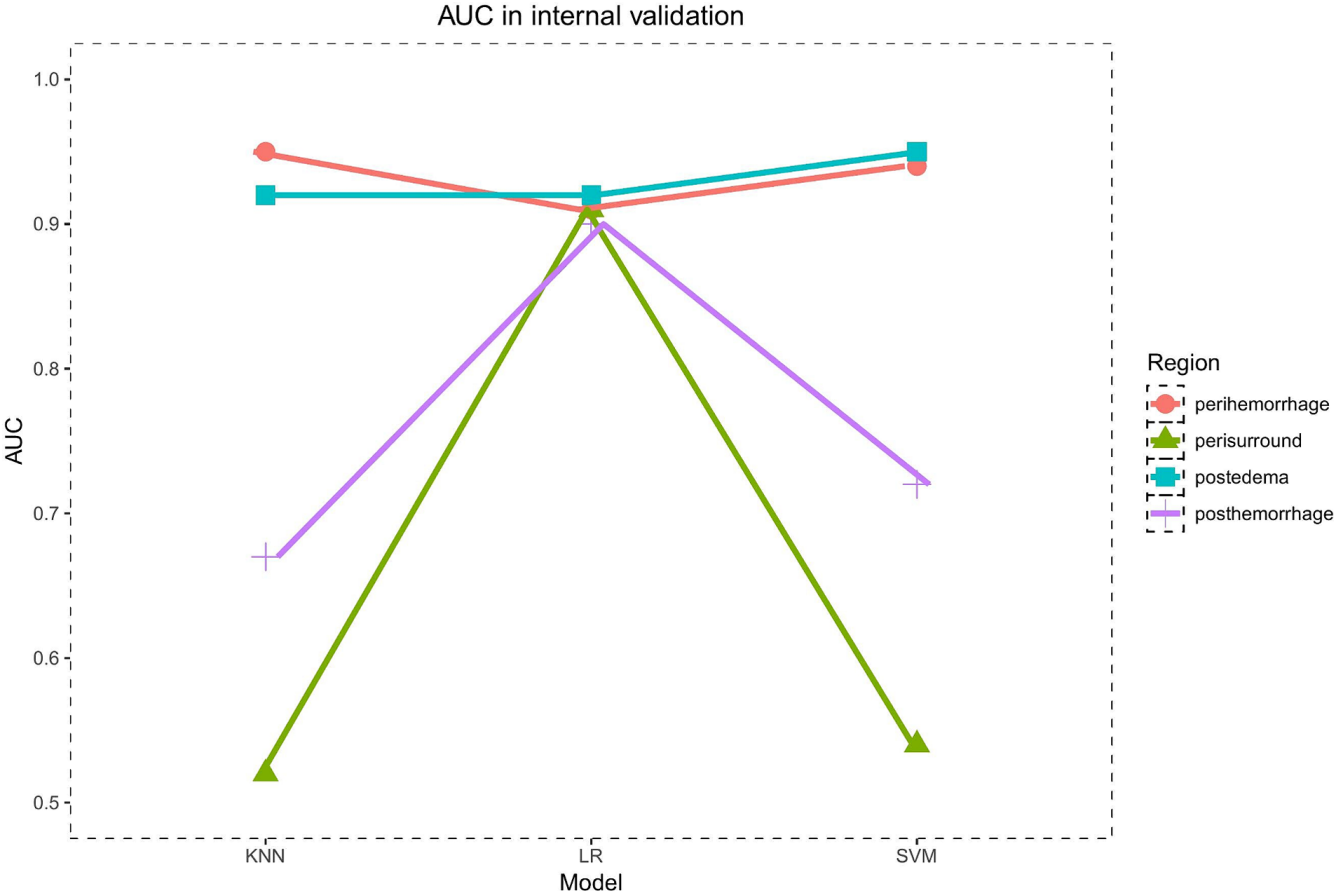
The AUC of models in internal validation.

In the internal validation set, the PRH-KNN, PRH-LR, and PRH-SVM models had AUC values of 0.95, 0.91, and 0.94, respectively (Figure 3A). The PRS-LR, PRS-KNN, and PRS-SVM models had AUC values of 0.91, 0.52, and 0.54, respectively (Figure 3B). The PSH-LR, PSH-KNN, and PSH-SVM models had AUC values of 0.90, 0.67, and 0.72, respectively (Figure 3C). The PSE-LR, PSE-KNN, and PSE-SVM models had AUC values of 0.92, 0.92, and 0.95, respectively (Figure 3D). Figure 4 shows the comparison of AUC values between the different machine learning models. The data indicate that the models constructed based on the perioperative hemorrhage area or post-edema area exhibited stable and relatively high performance.

### 3.4 Machine learning model in the external validation set

In the external validation set, the PRH-LR, PRH-KNN, PRH-SVM, PSE-LR, PSE-KNN, and PSE-SVM models exhibited AUC values of 0.92, 0.90, 0.89, 0.92, 0.91, and 0.88, respectively. Table 4 shows the AUC, sensitivity, specificity, and accuracy values of the machine learning model.

**Table 4:** AUC values of different models in the external validation set.

Model	AUC	Sensitivity	Specificity	Accuracy
PRH-LR	0.92	0.70	1	0.72
PRH-KNN	0.90	0.70	0.95	0.79
PRH-SVM	0.89	0.7	1	0.55
PSE-LR1	0.92	0.7	1	0.79
PSE-KNN1	0.91	0.70	0.95	0.79
PSE-SVM1	0.88	1	0.63	0.72

AUC, area under the curve; PRH, perioperative hemorrhage area; PSE, postoperative edema area; KNN, k-nearest neighbor classification; LR, logistic regression; SVM, support vector machine.

## 4 Discussion

Hypertensive ICH predominantly occurs in the basal ganglia, and patients with basal ganglia cerebral hemorrhage have high disability and mortality rates. With the advancement of minimally invasive surgery, the application of endoscopy in ICH surgery has increased. Endoscopy has shown great advantages over traditional craniotomy, including less trauma and a shorter operative time. Multiple studies (14) have demonstrated the heterogeneity of hemorrhages, which can predict ICH expansion. Similarly, research has shown that postoperative edema significantly affects early postoperative functional recovery. Therefore, this study used pyradiomics to quantitatively extract radiomic features from CT scans of different regions in patients and constructed corresponding machine learning models to accurately predict patient prognosis. This approach aims to guide neuroendoscopic interventions for basal ganglia cerebral hemorrhage and has substantial clinical relevance.

Radiomics can capture features that are difficult to observe with the naked eye and provide quantitative data for subsequent analyses. Morphological features primarily represent parameters, such as the size of the region of interest. Histogram features reflect the distribution of gray-level values of the hemorrhage or edema. The GLCM captures spatial relationships between pixels or voxels with predefined distances in different directions, capturing pairs of pixels or voxels with predefined grayscale intensities. The GLRLM primarily reflects the spatial distribution of pixels with the same grayscale level in one or more directions in the two-dimensional or three-dimensional space. The GLSZM reflects the relationship between the number of groups of adjacent pixels or voxels with the same grayscale level. The NGTDM quantifies the sum of the differences between the grayscale levels of pixels or voxels and their neighboring pixels or voxels within a predefined distance. The GLDM represents the grayscale relationships between the central pixels or voxels and their surroundings. Previous studies have suggested that the heterogeneity of hemorrhage and edema greatly affects prognosis. Therefore, we used pyradiomics to extract quantitative data on hemorrhage and edema. Our study provided a more comprehensive, accurate, and objective assessment of the impact of hemorrhage and edema on prognosis.

Currently, there are many prognostic models for intracerebral hemorrhage, such as the ICH (15), max-ICH (16), modified ICH (17), ICH-FOS (18), and MIS scores (19). Among these, the MIS score was the only prognostic model for patients who underwent minimally invasive surgery. The MIS score, initially developed using only 104 patients, showed poor performance in predicting prognosis at 1 month after discharge, with an AUC of <0.7. Machine learning methods can be used to explore disease patterns from complex data. Multiple studies (20, 21) attempted to use machine learning methods to construct models that can accurately predict the prognosis of patients with ICH. In this study, we constructed 12 models based on radiological and radiomic features from different regions, most of which can accurately predict the prognosis of patients with basal ganglia cerebral hemorrhage. The models constructed based on the perioperative hemorrhage area or postoperative edema area exhibited an overall higher predictive performance than the other models, such as ICH score and FUNC score model. Study had demonstrated that the ICH score. The study (22) showed that the highest AUC of ICH score model and FUNC model in predicting the functional outcome at 6 months after discharge was 0.87 and 0.8, respectively. Furthermore, they were consistently validated in the external validation set, which accurately predicted the prognosis of patients with basal ganglia cerebral hemorrhage. This study also demonstrated a close correlation between perioperative hemorrhage, postoperative edema, and the prognosis of patients with basal ganglia cerebral hemorrhage.

The differing performance of models based on different brain regions (PRH, PRS, PSH, PSE) can be attributed to several factors, such as anatomical, functional and pathological variability. Different brain regions serve unique anatomical characteristics, distinct functions and pathology, which can lead to variations in imaging features. For example, perihematomal edema (PHE) following ICH may contribute to the blood–brain barrier dysfunction (23), ion pump dysfunction in endothelial cells (24), and hemoglobin cytotoxicity (25). PHE can be the new biomarker for predicting the brain injury in patients with ICH. In summary, the varying performance of models based on different brain regions reflects both anatomical and functional differences that impact feature extraction and predictive accuracy. Gaining insight into these differences improves the clinical interpretation of model outcomes and could aid in advancing personalized medicine, as well as in the discovery of novel therapeutic targets.

In summary, the prognostic prediction model developed in this study, which was based on radiomics and radiological features from different regions, showed high prognostic prediction performance for patients with basal ganglia cerebral hemorrhage who underwent neuroendoscopy. This model can serve as a valuable tool for doctors to evaluate patient prognoses and guide treatment decisions.

## 5 Limitations

Firstly, the study is the small sample size used for external validation, which raises the possibility that the model might be overfitted to the data. Expanding the sample size across multiple centers could further bolster the study’s conclusions. Secondly, this study does not compare the machine learning model to established prognostic scores for ICH patients.

## Data Availability

The original contributions presented in the study are included in the article/supplementary material, further inquiries can be directed to the corresponding authors.
